# Machine learning approaches for predicting protein-ligand binding sites from sequence data

**DOI:** 10.3389/fbinf.2025.1520382

**Published:** 2025-02-03

**Authors:** Orhun Vural, Leon Jololian

**Affiliations:** Department of Electrical and Computer Engineering, The University of Alabama at Birmingham, Birmingham, AL, United States

**Keywords:** protein-ligand binding sites, computational drug discovery, sequence-based methods, deep learning, binding prediction

## Abstract

Proteins, composed of amino acids, are crucial for a wide range of biological functions. Proteins have various interaction sites, one of which is the protein-ligand binding site, essential for molecular interactions and biochemical reactions. These sites enable proteins to bind with other molecules, facilitating key biological functions. Accurate prediction of these binding sites is pivotal in computational drug discovery, helping to identify therapeutic targets and facilitate treatment development. Machine learning has made significant contributions to this field by improving the prediction of protein-ligand interactions. This paper reviews studies that use machine learning to predict protein-ligand binding sites from sequence data, focusing on recent advancements. The review examines various embedding methods and machine learning architectures, addressing current challenges and the ongoing debates in the field. Additionally, research gaps in the existing literature are highlighted, and potential future directions for advancing the field are discussed. This study provides a thorough overview of sequence-based approaches for predicting protein-ligand binding sites, offering insights into the current state of research and future possibilities.

## 1 Introduction

Protein-ligand binding sites are specific regions on proteins where various ligands—including small organic molecules, peptides, nucleotides, and proteins—can attach or bind (Zhao et al., 2020). Although experimental laboratory methods identify these regions with the highest accuracy, they are generally costly and time-consuming (Sadybekov and Katritch, 2023). Therefore, computational approaches to drug discovery have become increasingly important. These computational methods offer distinct advantages by reducing costs and speeding up identifying and optimizing potential drug candidates (Gupta et al., 2021). Predicting protein-ligand binding sites is a critical component of computational drug discovery, essential for pinpointing viable drug targets and advancing the development of new therapeutics (Stank et al., 2016). Recent advancements in machine learning have significantly improved this field by introducing sophisticated computational techniques to analyze the complex interactions between proteins and ligands (Xia et al., 2024). While traditional methods based on geometry, energy, or templates have been successful, deep learning has recently achieved much better results (Gagliardi et al., 2022). Deep learning models can learn complex patterns directly from raw data and generalize better across diverse datasets. Protein ligand binding sites prediction in computational models is divided into two main categories, based on input type: structure-based and sequence-based (Gamouh et al., 2023; Hosseini et al., 2024).

**FIGURE 1:**
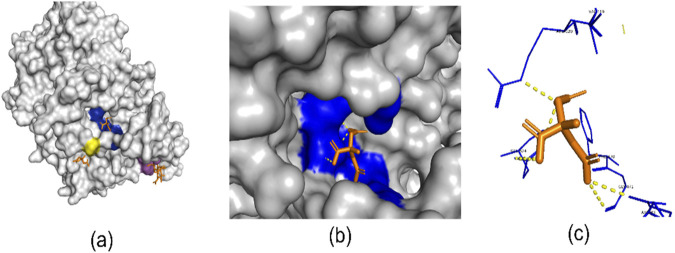
**(A)** Three binding site regions of 6Y3C protein in blue, yellow, and purple. **(B)** Close-up of a binding site with its ligand. **(C)** Ligand (orange) binding to a site. Generated with PyMol (Schrodinger, 2015).

Structure-based methods in computational drug discovery (SBDD) utilize detailed knowledge of the spatial information of proteins and integrate chemical properties using methods such as voxel-grid techniques (Sunseri and Koes, 2020). Figure 1 presents a 3D view of the 6Y3C protein and its associated ligands (Miciaccia et al., 2021). The number of protein-ligand binding sites on a protein can vary widely, depending on the specific protein and its function. In Figure 1A, the regions highlighted in yellow, blue, and purple represent protein-ligand binding sites. Figure 1B focuses on one of the binding sites shown in Figure 1A, offering a closer view of how the ligand interacts with the binding pocket. Figure 1C highlights the specific interactions between the ligand and the surrounding amino acid residues. In recent years, deep learning techniques used to identify these regions have often approached the problem as either image segmentation or object detection within structure-based frameworks. For instance, studies like RefinePocket (Liu et al., 2023), Kalasanty (Stepniewska-Dziubinska et al., 2020), PointSite (Yan et al., 2022), and DeepPocket (Aggarwal et al., 2021) use image segmentation techniques for binding site prediction, while RecurPocket (Li et al., 2022) and FRSite (Jiang et al., 2019) employ object detection techniques. Structure-based approaches depend on high-resolution 3D protein structures from X-ray crystallography or NMR spectroscopy (Maveyraud and Mourey, 2020). These methods face challenges such as reliance on accurate structures, static views of dynamic proteins, and high time and cost demands. AlphaFold (Abramson et al., 2024) has revolutionized the determination of 3D protein structures, significantly reducing reliance on experimental methods. However, drug discovery still primarily depends on 1D amino acid sequence data for critical tasks. Advancing approaches like AlphaFold requires a deeper understanding of the 1D sequence data used as input. This topic is further explored in the Discussion and Analysis section.

Sequence-based methods utilize one-dimensional (1D) amino acid sequence data as input. The 1D sequence is a direct representation of the protein’s genetic blueprint and is experimentally measurable with high reliability (Alfaro et al., 2021). Sequence-based methods are less computationally intensive, do not require high-resolution structural data, and can be applied to a wider variety of proteins, including those for which structural information is unavailable. There are many more known protein sequences than experimentally determined structures (Chelur and Priyakumar, 2022). The general process of sequence-based binding site identification begins with a given protein sequence as input, leading to the final prediction and evaluation. The first step is feature extraction, which is challenging due to the complexity and diversity of proteins. This involves converting linear sequence data into numerical vectors that accurately represent the protein’s functional and structural characteristics. Effective feature extraction is critical because the quality of the numerical representation directly impacts the performance of subsequent machine learning models. These techniques include binary representation, which encodes the presence or absence of specific amino acids; physicochemical representation, which considers the chemical and physical properties of amino acids; evolution-based representation, which leverages evolutionary information from multiple sequence alignments; and structure or machine learning-based representations, which use structural data or advanced algorithms to infer relevant features (Jing et al., 2019). Once the protein sequence is converted into a numerical format, it is ready for training with machine learning models using datasets with known binding sites. These datasets provide the necessary ground truth for model training and validation. The datasets most frequently employed in the literature are sc-PDB (Desaphy et al., 2015), COACH420 (Krivák and Hoksza, 2018), HOLO4k (Krivák and Hoksza, 2018), PDBBind (Liu et al., 2015), CSAR NRC-HiQ (Dunbar et al., 2013), UniProt (UniProt Consortium, 2015), Pfam (Finn et al., 2014), BioLip (Zhang et al., 2024), and PiSite (Higurashi et al., 2009). Each dataset has its unique characteristics and specific applications, contributing to the robustness and generalizability of the trained models. For instance, COACH420, derived from the COACH (Yang et al., 2013b) test set, is a widely recognized benchmark dataset that includes 420 protein-ligand complexes. Each complex consists of a single-chain protein intricately bound to a small molecule ligand. HOLO4K: A larger and more challenging dataset with 4,009 protein-ligand complexes. It includes multi-chain structures, offering a wider range of protein binding scenarios.

In this paper, we focus on sequence-based protein-ligand binding site prediction studies that employ machine learning techniques. As seen in Table 1, we have summarized these studies by focusing on their feature extraction techniques, and machine learning models. The Analysis and Discussion section provides a detailed evaluation of the machine learning models listed in Table 1, highlighting the strengths, limitations, and research gaps of sequence-based approaches. Additionally, potential future directions are outlined in the Future Directions section.

**TABLE 1:** Sequence-based machine learning models for predicting protein-ligand binding sites.

Model	Feature extraction methods	Machine learning model^a^	Dataset	Evaluation metric	Accuracy^b^	Year
SCRIBER (Zhang and Kurgan, 2019)	ASAquick, HHblits, ANCHOR, PSIPRED, AAindex	Logistic Regression	BioLip, UniProt, Pfam	MCC	0.230	2019
DeepCSeqSite (Cui et al., 2019)	PSSpred, Anglor, Jensen-Shannon divergence (JSD), Relative entropy	Deep Convolutional Neural Network	BioLip	MCC	0.496	2019
DELIA (Xia et al., 2020)	PSI-BLAST, HHblits, SCRATCH-1D, S-SITE	ResNet + BiLSTM	BioLip, ATPBind	MCC	0.469	2020
HoTs (Lee and Nam, 2022)	1D-CNN, hierarchical recurrent neural network	CNN + Transformers	scPDB, PDBbind, COACH420, HOLO4k	Top-n success rate (%)	66.3 ± 0.9	2022
Birds (Chelur and Priyakumar, 2022)	DeepMSA, PSIPRED, SOLVPRED	ResNet	scPDB	MCC	0.568	2022
T5 GAT Ensemble (Gamouh et al., 2023)	ProtT5	Graph Neural Network + Attention	BioLip, RCSB	MCC	0.592	2023
LaMPSite (Zhang and Xie, 2023)	ESM-2, RDKit	Pooling + Clustering	scPDB, COACH420	Top-n success rate	66.02	2023
Pseq2Sites (Seo et al., 2024)	ProtTrans	CNN + Attention	COACH420, HOLO4k, CSAR	Top-n success rate	96.8	2024
Seq-InSite (Hosseini et al., 2024)	ProtT5, MSA	MLP + LSTM	PiSite	MCC	0.462	2024

^a^ The Machine Learning Model column catalogs foundational models that constitute the core framework of the research presented, although the architecture of these studies may incorporate additional models.

^b^ The reported results are sourced from their own publications. Please note that direct comparisons between these values may not be valid due to differences in methodologies, preprocessing steps, and testing datasets. If separate results were provided for each ligand type, their average was calculated.

## 2 Sequence-based computational methods

Proteins are composed of a set of amino acids, each represented by a unique symbol (e.g., “A” for Alanine, “G” for Glycine). Similar to human language, which consists of sequences of words that convey meaning, protein sequences are structured in specific patterns that hold significant biological information. To analyze these sequences, feature engineering techniques are employed to derive meaningful attributes from the data. Machine learning models are then trained on these features to predict protein-ligand interactions or other relevant biological properties.

### 2.1 Feature engineering

Sequence-based methods leverage sequence data to capture biochemical and biophysical properties without direct 3D structural information. Multiple review papers provide a detailed overview of embedding approaches for protein sequence-based structures (Jing et al., 2019; Ibtehaz and Kihara, 2023; Villegas-Morcillo et al., 2022; Zhang and Liu, 2019; Hoksza and Gamouh, 2022; Tran et al., 2023). Embedding methods have been categorized in various ways across different studies. Jing et al. (2019) classified these methods into five distinct categories based on their information sources and methodologies: binary encoding, physicochemical properties encoding, evolution-based encoding, structure-based encoding, and machine-learning encoding. We categorize embedding methods into two groups: traditional embedding methods and machine learning-based embedding methods.

Transformer-based models (Vaswani et al., 2017) have gained popularity for applying linguistic analogies to protein sequences. For example, ProtTrans (Elnaggar et al., 2021), ESM-1b (Rives et al., 2021), and ESM-MSA (Rao et al., 2021) are transformer-based protein language models used for feature extraction. ProtTrans includes models like ProtBert and ProtT5, leveraging the transformer architecture to process large-scale protein datasets and produce sequence embeddings. ProtBert has 420 million parameters and was trained on 2 billion protein sequences. ESM-1b employs a transformer-based architecture to generate embeddings for protein sequences and has been trained on 250 million protein sequences. ESM-MSA is another protein language model that uses multiple sequence alignments (MSAs) from UniRef50 (Suzek et al., 2007) as input, interleaving row and column attention. It is trained on 26 million MSAs. Other popular advanced embedding methods for protein sequences are ProtVec (Asgari and Mofrad, 2015), SeqVec (Heinzinger et al., 2019), and UniRep (Alley et al., 2019). ProtVec uses the skip-gram-based Word2Vec model (Mikolov et al., 2013) to treat amino acid k-mers like words. It is trained on a corpus of 546,790 sequences obtained from Swiss-Prot (Boutet et al., 2007). SeqVec uses the Embeddings from Language Models (ELMo) (Sarzynska-Wawer et al., 2021) approach, which generates context-aware embeddings by considering the surrounding amino acids in a sequence. UniRep, based on a multiplicative Long Short-Term Memory (mLSTM) model (Krause et al., 2016), captures essential biochemical properties by predicting the next amino acid in a sequence and is trained on approximately 24 million protein sequences from UniRef50.

In addition to these protein language models, various other methods can be employed to create feature maps from protein sequences. These techniques include 1D-CNN, calculating relative solvent accessibility (RSA), position-specific score matrix (PSSM), secondary structure (SS), token embeddings, segment embeddings, one-hot encoding, conservation scores (CS), amino acid composition (AAC), physiochemical properties, and more (Laine et al., 2021; Guo et al., 2021; Raj and Chandra, 2024). Many specialized tools and software have been developed to calculate these features, enabling the generation of comprehensive feature maps from protein sequences.

### 2.2 Methodological approaches

Table 1 lists studies that focus on sequence-based protein binding site prediction. In this section, we provide an overview of each model included in Table 1, highlighting the feature extraction techniques employed, the specific machine learning algorithms applied.

SCRIBER (Zhang and Kurgan, 2019) converts input protein sequences into profiles representing structural, evolutionary, and physicochemical properties. These profiles include relative solvent accessibility (RSA) values predicted by ASAquick (Faraggi et al., 2014), which calculates solvent accessibility scores using only sequence-based features without relying on 3D protein structures and predicts the ASA for each residue based on encoded sequence features. Other features include evolutionary conservation values from HHblits (Remmert et al., 2012), relative amino acid propensity (RAAP) scores, protein-binding disorder from ANCHOR (Dosztányi et al., 2009), secondary structure from PSIPRED (Buchan et al., 2013), a sequence-based tool. Physicochemical properties (charge, hydrophobicity, and polarity) from the AAindex resource (Kawashima et al., 2007). SCRIBER employs a logistic regression model (Cramer, 2002) to predict protein-binding residues. SCRIBER processes a protein in approximately 45 s, significantly faster than PSI-BLAST, which takes 194 s, and PSI-BLAST combined with SANN (Joo et al., 2012), which requires 246 s.

DeepCSeqSite (Cui et al., 2019) leverages a Deep Convolutional Neural Network along with position-specific score matrix (PSSM), relative solvent accessibility (RSA), and secondary structure (SS) anticipated through PSSpred (Yan et al., 2013). RSA, a numeric value (often between 0 and 1), indicates how much of a residue’s surface is solvent-exposed versus buried. PSSpred uses neural networks to predict secondary structure elements, such as alpha-helices, beta-sheets, and coils, directly from sequence data. These elements, combined with positional embeddings, are used to build a detailed feature map from the protein sequence. To further enhance prediction accuracy, additional features such as conservation scores—calculated via Jensen-Shannon divergence (JSD) and relative entropy—residue type and dihedral angles, with predictions made by ANGLOR (Wu and Zhang, 2008), are incorporated.

DELIA (Xia et al., 2020) predicts protein–ligand binding residues using a hybrid model of convolutional neural networks (CNNs) (LeCun and Bengio, 1995) and bidirectional long short-term memory networks (BiLSTMs) (Schuster and Paliwal, 1997). It processes both 1D sequence feature vectors and 2D distance matrices to analyze amino acid sequences alongside protein spatial structures. DELIA utilizes sequence-based insights by integrating PSSMs from PSI-BLAST for evolutionary insights, fast and accurate evolutionary data from HHblits, secondary structure, and solvent accessibility predictions from SCRATCH-1D (Cheng et al., 2005), as well as binding propensities from S-SITE (Yang et al., 2013a). The SCRATCH software generates predictions for secondary structure and solvent accessibility using the amino acid sequence provided.

HoTS (Lee and Nam, 2022), employs a hierarchical recurrent neural network and 1D-CNN for protein sequence embedding to predict binding regions and drug–target interactions. HoTS leverages both CNN and transformer-based models, utilizing CNN layers to identify sequential motifs and transformers to model interdependencies. It also employs fully connected layers for accurately predicting binding regions.

Birds (Chelur and Priyakumar, 2022), utilizes a ResNet (He et al., 2016) architecture to predict a protein’s binding site based on the protein’s sequence information. This study employs a variety of techniques to extract information from protein sequences and construct a feature map, including token, positional, and segment embeddings, as well as multiple sequence alignments (MSAs) from DeepMSA (Zhang et al., 2020). From these MSAs, the position-specific score matrix (PSSM), Secondary Structure (SS), and Information Content (IC) were derived. Additionally, the Relative Solvent Accessibility (RSA) of each amino acid was determined by SOLVPRED from MetaPSICOV 2.0 (Jones et al., 2015).

T5 GAT Ensemble (Gamouh et al., 2023) predicts protein ligand binding sites with a hybrid approach combining sequence and structure data. This approach incorporates protein language models (pLMs) for sequence analysis and Graph Neural Networks (GNNs) (Scarselli et al., 2008) for structural insights, utilizing ProtT5-XL-UniRef50 (Elnaggar et al., 2021) to generate amino acid sequence embeddings. These embeddings serve as node features in the protein graph. The construction of the protein graph leverages the Python Deep Graph Library (DGL) (Wang et al., 2019), facilitating a sophisticated approach to modeling protein structures. In this graph, nodes are designated for individual residues, and edges define the spatial proximity between these residues. To determine the most suitable architecture, they tested two well-known GNN designs: the Graph Convolutional Network (GCN) (Kipf and Welling, 2016) and the Graph Attention Network (GAT) (Veliˇckovi´c et al., 2017).

LaMPSite (Zhang and Xie, 2023) predicts ligand binding sites using protein sequences and ligand molecular graphs. This approach incorporates residue-level embeddings from the ESM-2 protein language model (Lin et al., 2023) for proteins and atom-level embeddings from a graph neural network for ligands. Additionally, LaMPSite employs a pooling module to aggregate interaction embeddings, simplifying them to generate a residue-specific score. Then it clusters residues using the protein contact map, ranking these clusters to pinpoint binding sites. Current clustering and filtering processes typically yield one binding site per prediction, which may limit the identification of multiple or cryptic binding sites.

Pseq2Sites (Seo et al., 2024) uses ProtTrans, a transformer-based model, to extract amino acid-level embeddings for protein sequence analysis. Subsequently, 1D-CNNs were utilized to extract local features from the resulting embedding sequence, followed by the application of methods employing position-based attention mechanisms to capture long-distance contextual information.

Seq-InSite (Hosseini et al., 2024) utilizes ProtT5 and MSA-transformer embeddings to predict protein interaction sites from sequence data. Its architecture employs ensemble learning techniques, integrating a Multi-Layer Perceptron (MLP) and a Long Short-Term Memory (LSTM) (Hochreiter and Schmidhuber, 1997) network. While Seq-InSite predicts a broad range of protein interaction sites, including protein-ligand binding sites.

Overall, accurate prediction of protein-ligand binding sites is a crucial step in the drug discovery pipeline. Beyond theoretical predictions, these methods provide actionable insights that support drug target identification, lead optimization, and ligand design. Once protein binding sites are identified, these predictions lead to a variety of applications, including virtual screening (Kimber et al., 2021), studying off-target effects (Rao et al., 2023), predicting druggability scores (Raies et al., 2022), protein function prediction (Kulmanov and Hoehndorf, 2020), assessing mutation impacts (Sun et al., 2021), and pose prediction (Wang et al., 2022), among others.

## 3 Analysis and discussion

This section discusses four main topics: advancements in extracting features from protein sequences, the limitations of sequence-based methods with an analysis of the approaches listed in Table 1, the advantages of hybrid methods that combine sequence- and structure-based techniques, and a review of the datasets used for testing, as well as tools like AlphaFold that are employed for protein folding predictions. Each topic highlights critical aspects of the methodologies and their contributions to improving protein-ligand binding site predictions.

The models in Table 1 demonstrate a broad range of feature extraction techniques, spanning traditional evolution- and structure-based encodings to advanced protein language models (pLMs). 1D-CNNs are effective at extracting local motifs from protein sequences but may lose global context when motifs are spread across non-consecutive regions (Lee and Nam, 2022). PSSMs, a cornerstone of traditional methods, remain critical for capturing evolutionary information, with their removal causing significant performance drops (Chelur and Priyakumar, 2022). Relative solvent accessibility (RSA) and secondary structure elements add structural insights, but their impact on performance is less pronounced than that of embeddings or PSSMs (Chelur and Priyakumar, 2022). Secondary structure features and predicted dihedral angles provide structural context, with dihedral angles offering more fine-grained information; however, these features may also introduce noise (Cui et al., 2019). Protein language models, such as ProtT5-XL, offer significant advantages in terms of processing speed, generating embeddings for a human protein in as little as 0.12 s (Elnaggar et al., 2021). This efficiency is essential when analyzing extensive datasets with millions of sequences, allowing for high accuracy without reliance on traditional, computationally intensive evolutionary steps. ProtT5-XL embeddings, for example, deliver high accuracy and rich information, outperforming alternatives such as MSA-transformer embeddings in predictive tasks (Hosseini et al., 2024). Protein language models (pLMs) tend to be less effective for proteins that are rare or underrepresented in training datasets. However, pLMs perform best with well-represented proteins, and challenges remain in predicting binding sites for rare or novel proteins due to limited sequence data representation. As shown in Table 1, studies T5 GAT Ensemble, LaMPSite, Pseq2Sites, and Seq-InSite, which utilize pLMs extraction methods, demonstrate promising results compared to other studies listed in Table 1 that use traditional feature extraction methods.

One key advantage of sequence-based methods is their computational efficiency. For instance, on the well-known COACH420 dataset, sequence-based protein-ligand binding site prediction methods achieved significantly faster execution times: Pseq2Sites completed predictions in 1.07 s, Birds in 3.97 s, DeepCSeqSite in 11.13 s, and HoTs in 51.84 s. In contrast, structure-based methods were considerably slower, with DeepPocket taking 894.28 s, DeepSurf 2436.76 s, and P2Rank 914.61 s (Seo et al., 2024). Although sequence-based methods are computationally efficient, they lack the spatial context needed to identify complex binding interactions, such as those involving residues across multiple protein chains. By analyzing each chain individually and then combining the results, traditional sequence-based methods often miss critical relationships, limiting their accuracy in predicting binding sites. The studies in Table 1 highlight distinct characteristics of various models. For instance, SCRIBER incorporates over 1,000 input features and relies on feature elimination techniques to manage complexity, though it remains susceptible to overfitting. SCRIBER reported a Matthews correlation coefficient (MCC) (Chicco and Jurman, 2020) of 0.23. DELIA, on the other hand, is tailored for specific ligand types, which enhances predictive accuracy for those interactions but limits its applicability to general protein-ligand binding site prediction. DELIA achieved an average MCC of 0.469, which was derived from results across five different ligand types. Attention-based models like HoTS and Pseq2Sites excel at capturing both local interactions and long-range dependencies within sequences, making them effective for understanding complex sequence patterns. In the Pseq2Sites study, Pseq2Sites demonstrated a 96.8% success rate on the COACH test dataset, calculated as the number of correctly identified pockets divided by the total number of pockets. Additionally, the study reported success rates for other models, with HoTS achieving 14.3% and Birds reaching 70%, highlighting the comparative performance within the same evaluation framework. Seq-InSite achieved an MCC value of 0.462 on the Dset_448 dataset, which focuses on ligands that are not proteins. However, sequence-based models still struggle to fully capture inter-chain interactions, which are critical for predicting functional binding sites in multimeric proteins. Sequence-based approaches are generally less effective in identifying allosteric binding sites, which are often located far from the active site and can be missed without considering the protein’s full 3D structure (Xia et al., 2024).

Hybrid approaches, which integrate both sequence-based and structural features, have emerged as powerful strategies to enhance the accuracy of protein function prediction tasks. The T5-GAT Ensemble, a hybrid model, combines sequence and structural features of proteins. While the sequence-based MLP model achieves an MCC of 0.54, the hybrid model improves this to 0.59 by incorporating structural features. Similarly, DELIA, tested on five ligand types, demonstrated that the hybrid architecture outperformed sequence-based models in MCC scores for all ligand types. Another method, LaMPSite, predicts ligand binding sites by utilizing both protein sequences and ligand molecular graphs. The ablation study for LaMPSite indicates a decrease in accuracy when the interaction module, which combines the benefits of both methods, is omitted. For this study, the reported success rate in terms of DCA (Distance Cutoff Accuracy) is 66.02%.

The choice of datasets in protein-ligand binding site prediction plays a crucial role in developing and evaluating computational models. To ensure fair testing, addressing data leakage is essential, especially the similarity between training and test datasets. For instance, LaMPSite excludes scPDB structures with more than 50% sequence identity or 0.9 ligand similarity and removes proteins from COACH420. Pseq2Sites takes additional steps by using unseen test datasets and filtering proteins with ≤40% structural similarity for unbiased evaluation. Studies like HoTS further promote fair analysis by reporting results at various similarity thresholds.

Protein folding software such as AlphaFold can facilitate hybrid approaches, certain limitations persist. AlphaFold2 (AF2) relies on patterns extracted from known protein folds rather than understanding the physical and chemical basis of proteins (Agarwal and McShan, 2024). The experimentally determined 3D structural dataset is limited to fewer than 300,000 structures, compared to the billions of protein sequences available in public repositories. AlphaFold 3 (AF3) builds on the evoformer architecture from AF2, incorporating a diffusion network that refines a cloud of atoms iteratively to generate highly accurate protein structures. AF3 can predict heme-binding sites; however, its reliance on structurally similar proteins in its training data limits its effectiveness for less-represented or novel protein sequences (Kondo and Takano, 2024). AF3 struggles to accurately predict ligand-binding poses, particularly for complex ligands such as peptides, ions, and non-standard molecules (He et al., 2024). Additionally, the lack of support for user-defined ligands and a broader range of ligand types further restricts AF3’s applicability in practical drug discovery efforts. Single changes in the sequence (e.g., point mutations) can significantly alter a protein’s function or cause misfolding. AF2 is not trained to predict the effects of mutations on protein structure or stability (Agarwal and McShan, 2024; Pak et al., 2023). AF3 has limitations in stereochemistry, hallucinations, dynamic behavior, and accuracy for specific targets (Abramson et al., 2024). The Predicted Local Distance Difference Test (pLDDT) serves as a confidence metric in AF2 and AF3 for evaluating the reliability of protein structure predictions. However, high pLDDT values or low Predicted Aligned Error (PAE) scores do not necessarily ensure alignment with experimental structures (Carugo, 2023; Buel and Walters, 2022).

Overall, the paper highlights the strengths and limitations of both 3D and 1D approaches, concluding in the discussion section that hybrid methodologies represent a promising direction for future research.

## 4 Future directions

Future advancements in protein binding site prediction are likely to focus on integrating sequence-based and structure-based data to improve model accuracy, particularly for complex binding sites that depend on 3D spatial context. Hybrid models that combine these two types of data show promise in addressing limitations of sequence-only methods, such as identifying distant allosteric sites or inter-chain interactions. Another promising direction involves the development of transformer-based models specifically tailored for protein-ligand interactions, utilizing advanced embeddings to capture intricate sequence patterns and dependencies. Recently, GPT-based (Brown et al., 2020) studies have emerged in protein engineering, harnessing protein sequence data and the capabilities of large language models (LLMs) rooted in natural language processing (NLP). These advancements emphasize the need for a deeper understanding of protein sequence data, improving its representation, and designing deep learning architectures to align with these enhancements. Reviews like ours, which focus on sequence-based protein structures, are expected to make valuable contributions to the development of these tools. To further advance the field, it will be critical to enhance the adaptability of protein language models (pLMs) for underrepresented or rare proteins. This could be achieved by expanding training datasets or developing adaptive embedding methods. Additionally, collaboration across computational, experimental, and industrial fields will be essential for validating and refining these models. Such efforts aim to improve generalizability and optimize predictive tools for specific therapeutic targets, ultimately accelerating advancements in computational drug discovery.

## 5 Conclusion

The prediction of protein-ligand binding sites is crucial for advancing drug discovery and development, as it enables the identification of potential drug targets and the design of more effective therapeutics. Accurate prediction methods can significantly streamline the drug discovery process, reducing the time and cost associated with experimental validation. Our study reviews various sequence-based approaches for predicting protein-ligand binding sites using machine learning techniques in computational drug discovery. Our examination explores the models, focusing on their embedding methods and deep learning architectures, and discusses the challenges and future directions associated with sequence-based methods. Our study aims to serve as a comprehensive guide for sequence-based prediction of protein-ligand binding sites, providing a thorough understanding of the existing literature within a single paper.
